# Optimization of Sperm Cryopreservation Protocol for Peregrine Falcon (*Falco peregrinus*)

**DOI:** 10.3390/ani10040691

**Published:** 2020-04-16

**Authors:** Beatriz Cardoso, Irene Sánchez-Ajofrín, Cristina Castaño, Olga García-Álvarez, Milagros Cristina Esteso, Alejandro Maroto-Morales, María Iniesta-Cuerda, José Julián Garde, Julián Santiago-Moreno, Ana Josefa Soler

**Affiliations:** 1 SaBio IREC (CSIC-UCLM-JCCM), ETSIAM, 02071 Albacete, Spain; beacardoso_14@hotmail.com (B.C.); garciaalvarez.olga@gmail.com (O.G.-Á.); jandromaroto@hotmail.com (A.M.-M.); m.iniestacuerda@gmail.com (M.I.-C.); Julian.garde@uclm.es (J.J.G.); anajosefa.soler@uclm.es (A.J.S.); 2 Department of Animal Reproduction, INIA, 28040 Madrid, Spain; cristina.castano@inia.es (C.C.); esteso.milagros@inia.es (M.C.E.); moreno@inia.es (J.S.-M.)

**Keywords:** cryopreservation, peregrine falcon, spermatozoa, freezing/thawing, cryoprotectant

## Abstract

**Simple Summary:**

The process of semen cryopreservation can have multiple advantages in an ex situ conservation program. However, there is a necessity to adapt the protocol to the specificity of each species. With that in mind, we aimed to optimize the sperm freezing/thawing process and study the effect of different cryoprotectants in the peregrine falcon.

**Abstract:**

Sperm cryopreservation is a complex process that needs to be adapted to wild and domestic avian species to ensure proper efficiency. Because of its accessibility, the peregrine falcon may be used as a good model for studying other raptor species. To find the most optimal cryopreservation protocol for peregrine falcon ejaculates, sperm parameters such as motility, viability, DNA fragmentation, acrosome integrity, and mitochondrial activity were analyzed under different conditions by varying the freezing method (slow freezing in straws vs. ultrarapid freezing in pellets), thawing conditions (37 °C for 30 s vs. 5 °C for 1 min), type of cryoprotectant (DMA vs. DMSO), and concentration of DMSO (4% vs. 8%). Results show that slow cryopreservation in straws yielded greater percentages (*p* < 0.05) of motile spermatozoa (22.5% ± 4.4% vs. 0.0% ± 4.1%), viable spermatozoa with intact acrosomes (84.6% ± 4.3% vs. 77.4% ± 4.3%), and spermatozoa with active mitochondria (41.0% ± 6.7% vs.12.8% ± 6.7%), compared with those obtained by the ultrarapid freezing in pellets. However, no differences were found between different thawing conditions. Moreover, all sperm motility parameters were greater (*p* < 0.05) when DMSO was used during freezing compared with DMA, although the use of 3% and 8% DMSO produced similar results. In conclusion, these results represent important progress in the study of falcon semen cryopreservation protocol, highlighting the crucial steps of the process and the most suitable conditions.

## 1. Introduction

At present, the number of species threatened with extinction is above 28,000, of which 14% correspond to avian species [1]. With fast changes currently occurring in the climate and habitats, thousands of species of wild animals are in danger of disappearing [1]. A global effort is being made to create a solution for potential emergencies that could lead to the rapid extinction of species. With that in mind, captive breeding and the exchange of individuals between zoos are common practices around the world. However, space is limited, and it is impossible to maintain significant numbers of each existent species. A viable presented solution is the creation of a germplasm bank that contains the sperm, oocytes, or even embryos of the largest possible number of species [2,3].

Since the storage of eggs or embryos from birds poses a difficult challenge because of their large size and complexity, sperm cells are a good alternative [4]. Sperm is fairly easy and inexpensive to acquire, and its cryopreservation is much more accessible compared with the oocyte/embryo, because of the small volume of water inside of the sperm [5]. Furthermore, existing technologies allow the semen of many animal species to be collected and used [6]. Frozen semen can be a very important part of ex situ conservation efforts, since it allows for the storage of genetic material from multiple species for a long period of time. Stored semen can be used in reintroduction projects or to enlarge the genetic pool of a fragmented population. It also allows for the selection of individuals of genetic interest and the exchange of samples between distant locations [7,8]. In birds, frozen semen could become an increasingly relevant tool, because captive breeding programs are often slowed down by incompatibility between individuals or by stress, leading to the absence of mating [9]. Cryopreservation of semen is a technique largely used in mammals, but, in birds, there are still some limitations. Avian spermatozoa are filiform shaped, which makes them more susceptible to injury from manipulation [10]. Additionally, they are highly sensitive to the changes in osmolarity that occur during the freezing/thawing process [11,12].

Large numbers of peregrine falcons (*Falco peregrinus*) are managed in commercial breeding programs and could be used as a model for other birds of prey, especially ones that have spermatozoa with similar characteristics. By studying cryopreservation in falcons, viable solutions can be applied not only to the growing market of falconry, but also to a variety of other birds of prey, including wild endangered ones [9,13]. 

The objective of this study was to evaluate the effect of different conditions during falcon semen cryopreservation on several sperm characteristics. For this purpose, the freezing/thawing protocol and the type and concentration of cryoprotectant utilized were examined. The ultimate goal was to develop and optimize sperm cryopreservation protocols for this species and to increase the success rate of artificial inseminations with frozen semen.

## 2. Materials and Methods 

### 2.1. Animals

Semen was collected from seven adult male European peregrine falcons (*Falcon peregrinus peregrinus*) twice a week from mid-March to mid-April of 2014 and 2019. The specimens were owned by Sevilla falcons a limited company located in Sevilla, Spain, that is dedicated to breeding and selling falcons. The breeder considered the donors to be healthy, and no health issues were reported in the donors that season. 

### 2.2. Semen Collection

Semen samples were collected by voluntary false copulation or, alternatively, by forced abdominal massage, adapted to this species. All samples were obtained by capillarity using glass capillaries (75 µL Globe Scientific Inc^®^, Paramus, New Jersey, USA) and then transferred into 1.5 mL Eppendorf^®^ microcentrifuge tubes (Eppendorf Ibérica SLU, San Sebastián de los Reyes, Madrid, Spain). After collection, samples were diluted 1:1 in the diluent Lake 7.1 (composition: 0.08 g of magnesium acetate, 0.128 g of tripotassium citrate, 1.52 g of sodium glutamate, 0.6 g of glucose, 3.05 g of BES, and 5.8 mL of sodium hydroxide, diluted in 100 mL of distilled water (370 mOsm/kg, pH = 7.1)). Then, they were transported at room temperature. In the laboratory, motility parameters, viability, and the percentage of spermatozoa with DNA fragmentation were immediately evaluated. Ejaculates from two individuals with similar qualities were pooled before the start of the freezing process. Experiments 1 and 2 were replicated three times. The experimental procedures, evaluation of sperm quality, and freezing/thawing protocols could differ between experiments, because the study was spread out over time.

### 2.3. Experimental Design

Experiment 1 was carried out to examine the behavior of falcon spermatozoa subjected to different methods of freezing (Experiment 1A) and thawing conditions (Experiment 1B). Spermatozoa extended in Lake 7.1 were diluted in 3% DMA and equilibrated at 5 °C for 10 min and subjected to slow freezing in straws or ultrarapid freezing in pellets. Slow freezing was performed in nitrogen vapors after transferring the sample to 0.25 mL straws. The straws were placed in a racket 5 cm above the surface of a liquid nitrogen bath for 10 min. With this method, the temperature decreased from 5 to −85 °C at a rate of 10 °C/min. Immediately after, they were plunged into liquid nitrogen to achieve a temperature of −196 °C. For ultrarapid freezing in pellets, the diluted semen (60 µL) was directly plunged into liquid nitrogen to form a pellet. Afterward, samples were thawed in a water bath at 37 °C for 30 s, and sperm variables were evaluated. In experiment 1B, spermatozoa that were slowly cryopreserved in nitrogen vapors were thawed in a water bath at 37 °C for 30 s or at 5 °C for 1 min.

Experiment 2 was carried out to evaluate the effects of different types (Experiment 2A) and concentrations (Experiment 2B) of cryoprotectants on sperm quality after thawing. Two cryoprotectants, DMA and DMSO, were added to the diluted samples to a final concentration of 3% and 8%, respectively. Samples were allowed to equilibrate for 10 min at 5 °C and then transferred to 0.25 mL straws. Since the present research study was performed over a long period of time, the freezing protocol used in this experiment differed from the previous one. For this experiment, straws were frozen in a controlled-rate freezer (ICECUBE 1810 Sy-Lab), using the following two-step freezing ramp described by [14]: first, the temperature was decreased from −5 to −35 °C at a rate of 7 °C/min; then, it was decreased from −35 °C to −140 °C at 60 °C/min. The straws were then thawed at 37 °C for 30 s. The cryoprotectant that presented the best results was chosen in the subsequent experiment (2B), in which half of the ejaculates were extended with 4% of DMSO and the other half with 8%. 

### 2.4. Sperm Evaluation

#### 2.4.1. Sperm Motility

Sperm motility was evaluated both subjectively (SM) and with computer-assisted semen analysis (CASA) using the Sperm Class Analyzer software (SCA^®^ 2002, Microptic, Barcelona, Spain). The computer running this system was coupled to a Nikon Eclipse model 50i phase-contrast microscope (Nikon Instruments Europe B.V., Izasa S.A., Barcelona, Spain; negative contrast mode). The percentages showing total (sperm having a curvilinear velocity >10 µm/s) and progressive motility (spermatozoa swimming forward quickly in a straight line) were recorded. Other recorded sperm movement characteristics were straight-line velocity (VSL), the amplitude of lateral head displacement (ALH), and beat-cross frequency (BCF). A progression ratio, expressed as a percentage, was calculated: straightness (STR = straight-line velocity (VSL)/average path velocity (VAP) × 100). CASA settings for motility were progressive motility >75% STR and circular movement <50% linearity (LIN = VSL/VCL × 100). A minimum of three fields and 500 sperm tracks were evaluated at a magnification of 10× for each sample (image acquisition rate 25 frames/s).

#### 2.4.2. Sperm Viability and Acrosome Status

In Experiment 1, cell viability and acrosome integrity were evaluated by flow cytometry (Cytomics FC500 flow cytometer, Beckman Coulter, Inc. USA). A staining solution was prepared by adding propidium iodide (PI; 12 µM) and peanut agglutinin (PNA)-conjugated FITC (FITC-PNA; 1 µg/mL) in a buffered pH solution containing 20 mM HEPES, 197 mM NaCl, 2.5 mM KOH, and 10 mM glucose. Samples were incubated in the dark for 10 min at a final concentration of 10^6^ spermatozoa/mL. For the analysis, forward-scatter light (FSC) and side-scatter light (SSC) dot plots were used to discard debris. This fluorescent technique was used to distinguish among four sperm populations: PNA-/PI-, considered viable cells with intact acrosomes; PNA-/PI+, considered dead cells with intact acrosomes; PNA+/PI+, considered dead cells with damaged acrosomes; and PNA+/PI-, considered viable cells with damaged acrosomes. In our study, we took into account the first population (PNA-/PI-). 

In experiment 2, cell viability was examined using a combination of fluorochromes SYBR-14 and PI. When conducting this procedure, 2 µL of SYBR-14 and 5 µL of semen (previously diluted 1:1 in Lake 7.1) were added to an Eppendorf^®^ tube containing 200 µL of buffer solution and incubated in the dark for 10 min at 5–10 °C. Immediately afterward, 1 µL of PI was added to the solution, followed by incubation at 5 °C for 2 min. A total of 100 spermatozoa were evaluated using a fluorescence microscope (Eclipse E200, Nikon, Tokyo, Japan) at 40× (wavelength: 450–490 nm). Spermatozoa stained green (no PI staining) were considered to be alive, while red-colored spermatozoa were considered to be dead (any red color means that the membrane is impaired and has lost function). The percentage of viable cells was recorded.

#### 2.4.3. Mitochondrial Status

In experiment 1A, JC-1 dye was used to study mitochondrial status. Samples containing 5 µL of semen and 1 µL of JC-1 (1.5 mM JC-1 in DMSO) were incubated for 30 min at 37 °C in a dark environment. After staining, samples were analyzed using a flow cytometer (Cytomics FC500, Beckman Coulter, Inc., USA). The percentage of cells with high membrane potential (active mitochondria) was recorded.

#### 2.4.4. DNA Fragmentation

In Experiment 2, the amount of sperm DNA fragmentation and apoptosis was determined by TUNEL assay using a commercially available kit (In Situ Cell Death Detection Kit, fluorescein, Roche Biochemicals, Basel, Switzerland). In this assay, the free 3-OH ends of DNA are labeled with fluorescein conjugated to dUTP by the enzyme terminal deoxynucleotidyl transferase (TdT). Briefly, 2–7 µL of semen was fixed with 4% (w/v) paraformaldehyde (100 µL) in PBS. The tube was centrifuged for 10 min at 210 rpm. Seventy-five microliters was removed from the top layer, and the sample was resuspended in the same medium. Glass slides were washed with detergent and water to prevent the sample from coming off at any step of the process. Circles were drawn with a glass marker and surrounded by liquid blocker. Ten microliters of the sample were placed in each circle, dried on a hot plate, and then permeabilized with 0.1% (v/v) Triton X-100 (20 µL) for 5 min at room temperature in a dark humidified chamber. After washing with PBS, 10 µL of the terminal deoxynucleotidyl transferase (TdT)–fluorescent-labeled nucleotide mix (91.7% of nucleotide buffer and 8.3% of TdT enzyme) was added to the slides, and the labeling reaction was carried out for 1 h in the dark in a humidified chamber at 37 °C. After labeling, slides were rinsed twice in PBS and then counterstained with 0.1 mg/mL Hoechst 33342 (20 µL) in a humidified chamber for 5 min at room temperature to visualize total DNA. For the final step, a drop of aqueous mounting medium (Fluoromont^®^, Sigma-Aldrich, MO, USA) was layered across each circle on the slides. At least 200 cells of each sample were analyzed with a fluorescence microscope (Eclipse E200, Nikon, Tokyo, Japan). Each spermatozoon was deemed to contain either normal (blue nuclear fluorescence due to Hoechst 33342) or fragmented DNA (red nuclear fluorescence). The final percentage of sperm with fragmented DNA was calculated by dividing the number of apoptotic cells (red) by the number of cells detected with Hoechst 33342 (blue) and referred to as % TUNEL positive.

### 2.5. Statistical Analysis

All statistical analyses were performed using the IBM 24.0 software package. Prior to data analysis, the assumption of normality was tested, and if not satisfied, log or arcsin transformation was used. The effect of the freezing and thawing methods and type and concentration of cryoprotectant were studied by factorial ANOVA. The dependent variables considered were sperm motility parameters, viability, mitochondrial activity, and DNA fragmentation. When ANOVA revealed a significant effect, values were compared by a Bonferroni test. A *p*-value ≤ 0.05 was considered statistically significant. Results are presented as the mean ± S.E.M.

## 3. Results

The mean values of sperm motility variables, sperm viability, and percentage of spermatozoa with fragmented DNA of fresh ejaculate are shown in Table 1.

### 3.1. Experiment 1: Effect of the Freezing Method and Thawing Conditions on Sperm Quality

**Figure 1:**
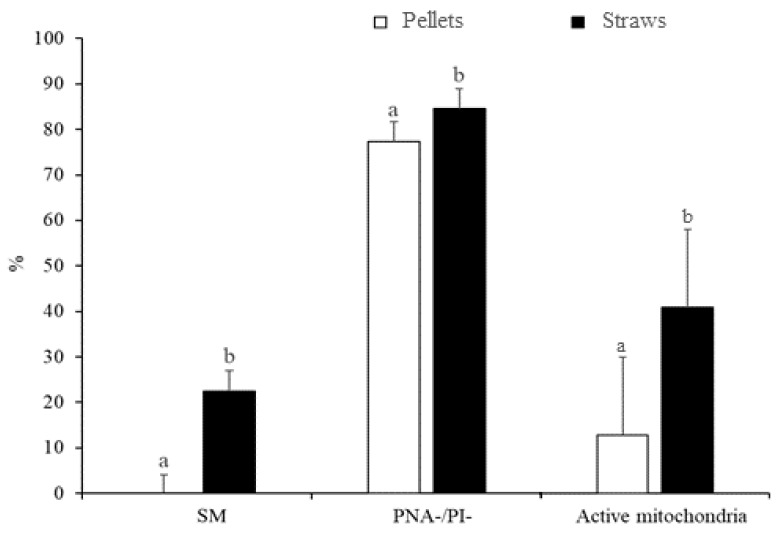
Characteristics of peregrine falcon sperm cryopreserved by slow freezing in straws and ultrarapid freezing in pellets: motile spermatozoa (SM), viable spermatozoa with intact acrosomes (PNA-/PI-), and spermatozoa with high membrane potential (active mitochondria). Results are expressed as the mean ± S.E.M. ^a,b^ Different letters indicate significant differences (*p* ≤ 0.05) between treatments.

The differences in sperm quality between the two freezing methods (slow freezing in straws and fast freezing in pellets) are indicated in Figure 1. The susceptibility to cryopreservation appeared to vary between treatments, since samples slowly cryopreserved in straws showed greater sperm quality after thawing than samples that underwent ultrarapid freezing in pellets (*p* < 0.05). Accordingly, the slow freezing method resulted in greater (*p* < 0.05) rates of SM (22.5% ± 4.4% vs. 0.0% ± 4.1%), PNA-/PI- (84.6% ± 4.3% vs. 77.4% ± 4.3%), and active mitochondria (41.0% ± 6.7% vs. 12.8% ± 6.7%) compared with ultrarapid freezing (Figure 1).

**Figure 2:**
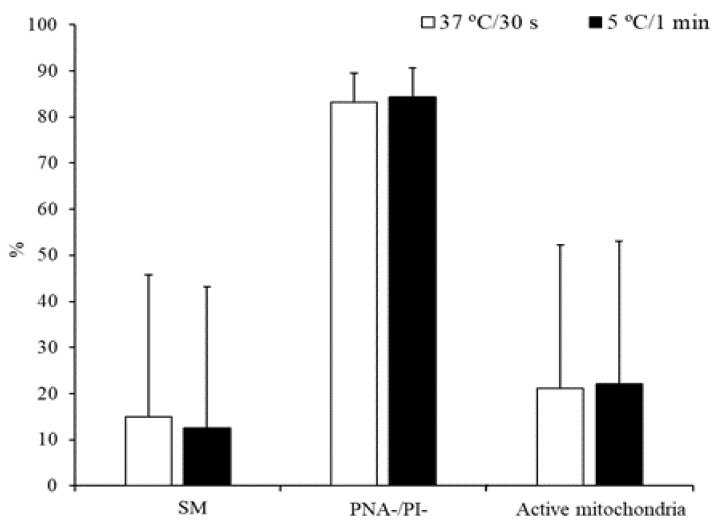
Characteristics of peregrine falcon sperm thawed at 37 °C for 30 s or 5 °C for 1 min: motile spermatozoa (SM), viable spermatozoa with intact acrosomes (PNA-/PI-), and spermatozoa with high membrane potential (active mitochondria). Results are expressed as the mean ± S.E.M.

On the other hand, no significant differences were detected for the above-mentioned parameters when sperm samples were thawed at 37 °C for 30 s or 5 °C for 1 min (Figure 2).

### 3.2. Experiment 2: Effect of the Type and Concentration of Cryoprotectant on Sperm Quality

**Figure 3:**
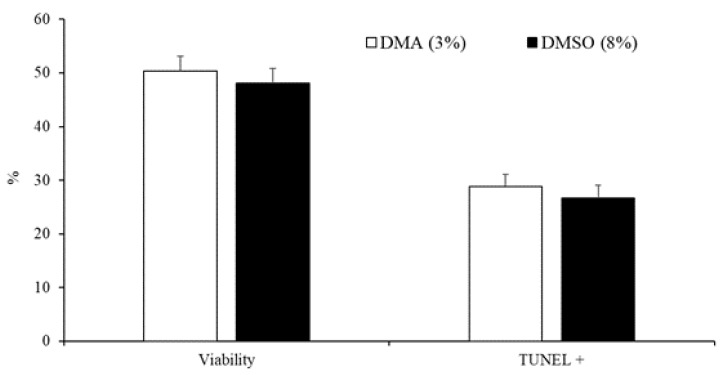
Sperm viability and spermatozoa with DNA fragmentation detected by TUNEL assay of samples cryopreserved in DMA (3%) or DMSO (8%). Results are expressed as the mean ± S.E.M.

The data on the effect of the type of cryoprotectant (3% DMA or 8% DMSO) on the motility, viability, and DNA fragmentation of peregrine falcon spermatozoa are presented in Table 2 and Figure 3. Ejaculates that were frozen in 8% DMSO had greater values for all motility variables compared with those in 3% DMA (Table 2). Nevertheless, there were no differences (*p* < 0.05) in the percentage of viable cells and spermatozoa with fragmented DNA between samples frozen with 3% DMA and those frozen in 8% DMSO (Figure 3).

**Figure 4:**
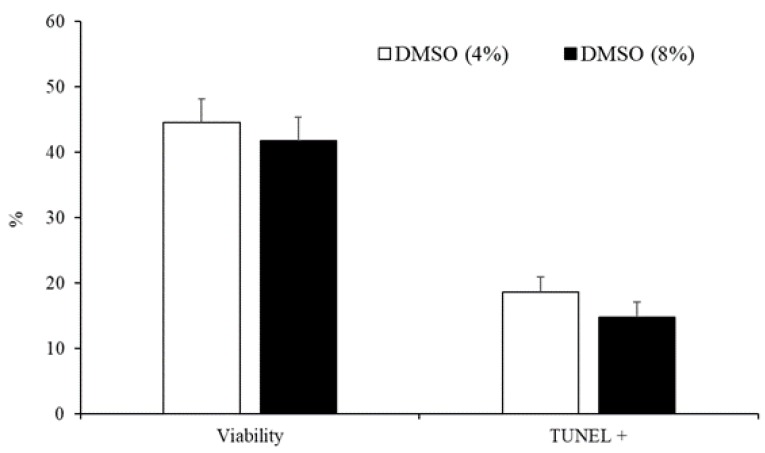
Sperm viability and spermatozoa with DNA fragmentation detected by TUNEL assay of samples cryopreserved with different concentrations of DMSO (4% or 8%). Results are expressed as the mean ± S.E.M.

The concentration of DMSO (4% vs. 8%) did not affect sperm motility parameters (Table 3) or the percentage of viable cells and spermatozoa with fragmented DNA (Figure 4). 

## 4. Discussion

Semen cryopreservation is a complex process with a number of unanswered questions, and this is particularly true in bird species [15]. There is a need to adapt the cryopreservation protocols for domestic species to wild species to improve the efficacy of the freezing process and to allow for its application in germplasm banks, reintroduction projects, and the breeding of domesticated falcons. In this study, samples obtained from peregrine falcons were used to compare different cryoprotectants, freezing techniques, and thawing conditions. 

Our results showed that the most critical steps of the cryopreservation process in this species were the freezing procedure and cryoprotectant used. Thus, the slow freezing method in straws showed better results in terms of sperm quality compared with ultrarapid freezing in pellets, which could potentially lead to better fertility outcomes. This was illustrated by a higher percentage of motile spermatozoa and viable spermatozoa with intact acrosomes and active mitochondria. This scenario agrees with previous studies that have reported better outcomes when sperm was preserved by slow freezing compared with those subjected to rapid cooling rates, as observed in several species, including turkeys (*Meleagaris gallopavo*), sandhill cranes (*Grus canadensis*), and roosters (*Gallus gallus domesticus*) [14,16]. Ultrarapid freezing in pellets might produce intracellular vitrification by drastically accelerating the cooling process, preventing the formation of intracellular ice crystals that can damage cells during the freezing/thawing process. To achieve a glass-like state, extremely high concentrations of cryoprotectants are required inside the cell. However, in birds, the size of the sperm head is much smaller than that in mammal species, and their cytoplasmic volume is also lower, which could hinder the cryoprotectant passage inside the sperm head [11,17]. Moreover, it is possible that vitrified samples could crystallize during the process of heating, or the formation of ice crystals in the extracellular milieu may occur even during ultrarapid freezing, thereby damaging the sperm membranes, as proposed by Bóveda et al. [18] in mammals. Therefore, although ultrarapid freezing in pellets has apparently great advantages, the poor results obtained suggest that the pellet method might not be the most suitable freezing approach for falcon spermatozoa. 

The time and temperature of the straw-thawing process did not have a significant impact on the motility and viability of sperm cells. This is in agreement with the study by Santiago-Moreno et al. [19], in which the thawing conditions had no influence on sperm quality in the gentoo penguin (*Pygoscelis papua*). However, in other species, such as black-footed penguins (*Spheniscus demersus*) and roosters, higher thawing temperatures had a more detrimental effect on spermatozoa [14,19,20]. Therefore, it seems that the thawing temperature may affect sperm quality characteristics in a species-specific manner. Nevertheless, more studies are necessary to conclude whether this step is, in fact, not critical during falcon sperm cryopreservation.

In reference to the type of cryoprotectant, our results showed that DMA had a considerably more damaging effect on motility parameters than DMSO. Although, in a general sense, DMSO is a less explored and used cryoprotectant, our findings also agree with those obtained in turkeys, whooping cranes (*Grus americana*), and white-naped cranes (*Antigone vipio*), in which better post-thaw sperm motility, sperm viability, and inner perivitelline membrane binding rates were obtained when DMSO was compared with DMA [21,22]. In contrast, Blanco et al. [16] reported an increase in sperm viability and fertility when DMA was used as a cryoprotectant in sandhill crane sperm. These inconsistent results might be explained by species-specific differences in the membrane composition and seminal plasma content. In domestic bird species, DMA is often recommended for sperm cryopreservation in pellets, and it yields better results when applied to protocols with rapid cooling rates [15,20,23]. In contrast, DMSO is better suited to slow freezing methods [24]. Additionally, low fertility outcomes have been observed when DMA/straw-based cryopreservation was compared with sperm cryopreserved with glycerol; this finding has been attributed to a negative interaction between the plastic straws and the cryoprotectant [23].

The impact of different DMSO concentrations was tested, and no significant differences were found in any of the motility and sperm quality parameters analyzed. The increase in the concentration of a cryoprotectant can have adverse effects for two reasons: the rise in osmolarity can lead to an osmotic shock, and the toxic effects of the chemicals can result in cell damage. DMSO is a permeating cryoprotectant that has two different effects, depending on the concentration used. In lower concentrations, it causes a membrane thinning effect that, in turn, facilitates the flow of water and cryoprotectant. In high concentrations, DMSO can form pores in the membrane, which may ultimately lead to the destruction of the entire cell [25]. A balance between the two effects, possibly achieved by concentrations of 8%–10%, was suggested as ideal in a simulation with artificial lipidic membranes [25]. Still, previous studies suggest that the tolerance of avian sperm to changes in cryoprotectant concentrations can be highly species-specific. In several studies comparing different concentrations of DMSO, optimal results were achieved with 4.8% in turkeys [26]; 6% in ring-necked pheasants (*Phasianus colchicus*), chickens [27], and sandhill cranes [28]; 8% in Indian red jungle fowl (*Gallus gallus murghi*) [23]; and 10% in ducks (*Anas platyrhynchos*) [29] and kestrels [30]. Previous studies that examined the effect of extreme osmolarities determined that wild avian species had a higher resistance to hyperosmolar conditions when compared with domestic ones [27,30,31]. These studies concluded that raptor species (eagles and falcons) exhibited extreme opposition to hyperosmolarity, possibly as a result of the sperm morphology, since spermatozoa of the peregrine falcon are longer in diameter compared with those in species such as poultry [31,32]. Furthermore, Blesbois et al. [33] postulated that differences between species of domestic birds in the initial spermatozoon membrane fluidity probably originated from variations in the cholesterol content of the membranes.

## 5. Conclusions

In summary, the freezing method and type of cryoprotectant utilized were identified as critical steps in the process of peregrine falcon sperm cryopreservation. The cryopreservation of peregrine falcon sperm by slow freezing in straws in combination with DMSO resulted in higher motility rates, although the thawing procedure and DMSO concentration had no effect on sperm quality. In addition, the results suggest that DMA is not ideal when slow cooling rates are used. Further artificial insemination studies are required to determine whether fertility rates are greater using slow sperm-freezing methods.

## Figures and Tables

**Table 1:** Sperm characteristics of fresh ejaculate diluted in Lake 7.1 for the peregrine falcon.

Parameter	Value
Concentration (× 10^6^/mL)	12.3 ± 1.3
Total motility (%)	73.8 ± 2.6
Progressive motility (%)	24.7 ± 4.0
Straight-line Velocity (VSL; µm/s)	27.0 ± 1.1
Straightness (STR; %)	82.6 ± 1.4
Amplitude of Lateral Head (ALH; µm)	2.1 ± 0.1
Beat-cross frequency (BCF; Hz)	9.8 ± 0.4
Viability (%)	82.3 ± 2.9
TUNEL + (%)	4.8 ± 1.5

TUNEL +: spermatozoa with fragmented DNA.

**Table 2:** Effect of type of cryoprotectant on motility parameters of peregrine falcon spermatozoa.

Semen Variables	DMA 3%	DMSO 8%
Motile spermatozoa (%)	4.2 ± 1.9 ^a^	13.9 ± 1.8 ^b^
Total motility (%)	7.7 ± 2.3 ^a^	21.1 ± 2.3 ^b^
Progressive motility (%)	1.1 ± 0.3 ^a^	2.1 ± 0.3 ^b^
Straight-line Velocity (VSL; µm/s)	7.7 ± 1.4^a^	12.9 ± 1.4^b^
Straightness (STR; %)	49 ± 6.4 ^a^	70.6 ± 6.2 ^b^
Amplitude of Lateral Head (ALH; µm)	0.3 ± 0.1 ^a^	0.8 ± 0.1 ^b^
Beat-cross frequency (BCF; Hz)	1.2 ± 0.5 ^a^	3.1 ± 0.5 ^b^

^a,b^ Different letters within rows reflect significant differences (*p* < 0.05) among treatments. Results are expressed as the mean ± S.E.M.

**Table 3:** Effect of DMSO concentration (4% vs. 8%) on sperm motility variables.

Semen Variables	DMSO 4%	DMSO 8%
Motile spermatozoa (%)	9.5 ± 1.9	8.1 ± 1.9
Total motility (%)	14.5 ± 2.7	12.6 ± 2.7
Progressive motility (%)	2.0 ± 0.5	1.6 ± 0.5
Straight-line Velocity (VSL; µm/s)	16.1 ± 2.9	13.2 ± 2.9
Straightness (STR; %)	77.8 ± 10.4	69.4 ± 10.4
Amplitude of Lateral Head (ALH; µm)	0.4 ± 0.2	0.4 ± 0.2
Beat-cross frequency (BCF; Hz)	2.5 ± 0.8	2.5 ± 0.8
